# Notes on *Scolytus* fagi Walsh 1867 with the ignation of a neotype, distribution notes and Key to *Scolytus* Geoffroy of America east the Mississippi River (Coleoptera, Curculionidae, Scolytinae, Scolytini)

**DOI:** 10.3897/zookeys.56.516

**Published:** 2010-09-17

**Authors:** Sarah M. Smith, Anthony I. Cognato

**Affiliations:** Department of Entomology, Michigan State University, 243 Natural Science, East Lansing, MI 48824, USA

**Keywords:** Scolytidae, Sbark beetle, Staxonomy, Nearctic

## Abstract

The identification of Scolytus fagi Walsh has been difficult because of the lack of diagnostic literature, the occurrence of several morphologically similar sympatric Scolytus species and the loss of the syntypes. In an effort to reduce taxonomic confusion, we designate a neotype for Scolytus fagi, redescribe the male and female, add new distributional records and create a key for the identification of eastern Scolytus species.

## Introduction

Specimens of Scolytus fagi Walsh have been rarely collected and within the past 140 years the species was only recorded from Illinois and Texas (Wood 1982). However, in 2009 specimens were collected in surprisingly large numbers from several locations in Pennsylvania. Initially, species identification of these specimens was difficult partially due to vague species descriptions, inadequate keys, a lack of illustrations and most importantly the loss of the syntypes (Walsh 1867, Blackman 1934, Wood 1982). Walsh (1867) described Scolytus fagi from a series of six syntypes collected from “southern Illinois” from what was presumably a beech tree (Fagus sp.). These specimens were stored in Walsh’s personal collection. Shortly after his death in 1869, the state of Illinois purchased his entire collection and moved it to the ‘fire-proof building’ of the Chicago Academy of Sciences (CASM). Unfortunately, the wing and nearly all of Walsh’s specimens were destroyed in the Great Chicago Fire of 1871 (Sheppard 2004). A few of Walsh’s Coleoptera and Lepidoptera specimens survived in the Chicago Academy of Sciences (Sheppard 2004), however the syntypes of Scolytus fagi were not among them (J. Colby, pers. comm.). These circumstances warrant the designation of a neotype to maintain nomenclatural stability and reduce taxonomic confusion with morphologically similar sympatric species such as Scolytus muticus Say and Scolytus quadrispinosus Say.

In this publication, we designate a neotype for Scolytus fagi, redescribe the male and female, add new distributional records and create a key for the identification of eastern Scolytus species.

## Materials and methods

Scolytus specimens were examined from the following collections (following Evenhuis 2009) for the creation of the key:

MCZMuseum of Comparative Zoology, Cambridge, MA (Phil Perkins)

MSUCAlbert J. Cook Arthropod Research Collection, East Lansing, MI (Gary Parsons)

NMNHNational Museum of Natural History, Washington, DC (Natalia Vandenberg)

SEMCSnow Entomological Museum, Lawrence, KS (Zack Falin)

UMMZUniversity of Michigan Museum of Zoology, Ann Arbor, MI (Mark O’Brien).

Scolytus specimens collected by United States Forest Service	 Early Detection and Rapid Response Program in Missouri and Pennsylvania were also examined as part of this study.

## Scolytus fagi Walsh

Scolytus fagi is known from a few specimens collected from Columbus, Texas, specimens from Illinois were difficult to locate. A single specimen of Scolytus fagi from Galesburg, Illinois was found in the collection of the MCZ (T.H. Atkinson, pers. comm.), and is here designated as the neotype. The specimen was examined by the authors and was chosen because it matches Walsh’s description, is from the same state as the type series and is in good condition.

Neotype. Male, vouchered in the MCZ and bearing the following labels:

1)	“Galesburg/Ill”

2)	“Liebeck/Collection”

3)	“S. /fagi/Walsh”

4)	“Scolytus fagi Walsh/Det. Atkinson 88”

### Redescription

**Figure 1.**
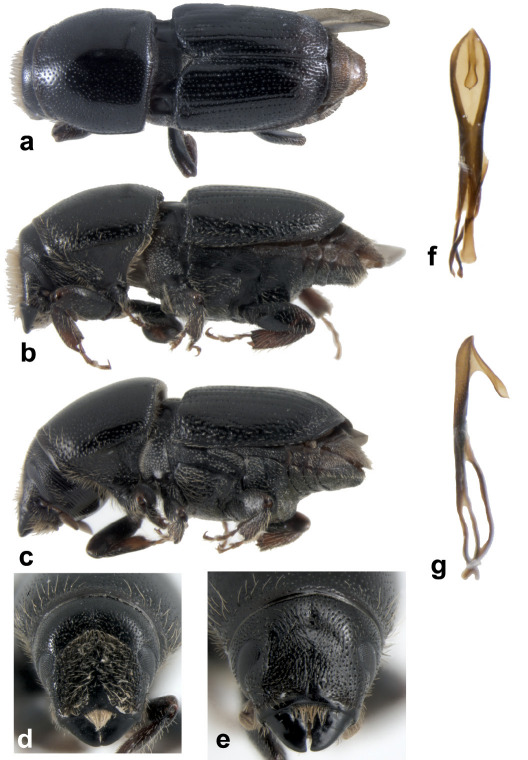
Scolytus fagi **a** male habitus dorsal **b** male habitus **c** female habitus **d** male frons **e** female frons **f** male genitalia, ventral view **g** male genitalia, lateral view.

Male: body length 3.5–6.0 mm long (x_= 5.0 mm; n = 20); 2.1–2.5 (x_= 2.37) times as long as wide. Color dark reddish brown to black. Dorsal habitus (Fig. 1a), lateral habitus (Fig. 1b).

Frons flattened, feebly concave, more strongly concave above epistoma and weakly concave above upper level of eyes, concave surface of frons punctate-granulate and densely covered with fine, long setae with apices directed toward the median line, basal and lateral margins of concavity with fewer, shorter, finer setae; median line devoid of granules, faintly aciculate and shining (Fig. 1d). Antennae dark reddish brown, club covered by short golden hair-like setae with two strongly procurved sutures.

Pronotum slightly longer than wide. Pronotum dark brown to black, margins reddish brown; pronotal surface shining, disk shallowly and minutely punctate, punctures on basal and its lateral sides larger and deeper; median line devoid of punctures on disk. Sparse, erect hair-like setae on apical and lateral margins of pronotum. Basal and lateral margins carinate, nearly straight.

Scutellum triangular, covered by fine recumbent golden hair-like setae and deeply set in the shagreened and subopaque scutellar impression. Elytra dark reddish-brown to black, slightly narrower than pronotum. Elytral strial punctures 2–3 times the size of those on interstriae, interstriae 2–2.5 times the width of striae; elytral surface shining, glabrous. Striae impressed, interstriae not impressed. Interstriae 9 and 10 and declivital interstriae covered with sparse and irregularly spaced setae. Elytral lateral edges feebly serrate, apex smooth, weakly emarginate.

Abdomen reddish-brown to black, surface of sternite 1, smooth, shining; sternites 2–5 shagreened, subopaque. Sternite 2 vertical (face at an angle of approximately 90º to first sternite) unarmed, coarsely punctured and covered with sparse setae. Apical margin of sternites 1–4 with a faintly raised margin. Sternite 5 longer than sternites 3 and 4 combined; apical fourth of sternite 5 subvertical, transversely impressed, more closely and coarsely punctured, moderately covered with abundant fine, hair-like setae each 2–3 times the length of setae on sternite 2. Genitalia (ventral view, Fig. 1f; lateral view, Fig. 1g).

Female (lateral habitus, Fig. 1c): similar to male except frons convex, faintly aciculate and devoid of granules, frons less abundantly covered by fine, long setae, basal and lateral margins with fewer, shorter, finer setae (Fig. 1e). Abdominal sternite 5 with a strongly elevated transverse subapical carina.

**Figure 2:**
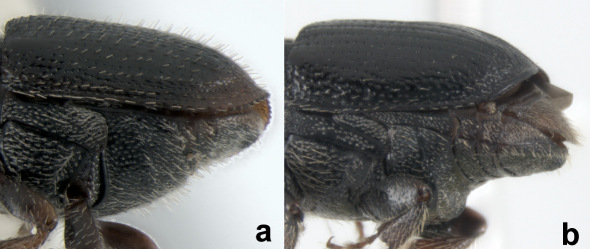
**a** Sternite 2 oblique (Scolytus rugulosus) **b** sternite 2 vertical (Scolytus fagi).

### Natural history

There is a single report of the life history by Packard (1890) in which Scolytus fagi was reported by Schwarz to colonize Celtis texana (=Celtis laevigata var. texana) in Texas and were “found boring in the solid wood in all stages… so numerous were the insects that the pattern of the larval burrow…was confused and undecipherable” (1890). Schwarz also reported that they did not appear to colonize healthy trees, but were very destructive to Celtis laevigata var. texana (1890).

In addition to Celtis laevigata, Scolytus fagi has been reported to colonize Celtis occidentalis (Cannabaceae), no locality given (Blatchley and Leng 1916; Chamberlin 1939), Celtis tenuifolia (Bright and Skidmore 1994) in Ontario, Fagus americana (Fagaceae) no locality given (Blatchley and Leng 1916; Chamberlin 1939), Fagus grandifolia, no locality given (Wood 1982), and Quercus sp. (Fagaceae) in Mississippi (Atkinson et al. 1991).

### Distribution

Scolytus fagi was known only from Illinois (Walsh 1867), Mississippi (Atkinson et al. 1991), Texas (Packard 1890), and southern Ontario (Bright and Skidmore 1994). Here we report new state records of Scolytus fagi from Kansas, Ohio and Pennsylvania. It is likely that Scolytus fagi occurs throughout the range of its hosts and the sparse locality records are due to the inadequate sampling.

#### Kansas

(vouchers deposited in the SEMC)

*Bourbon Co.*: Fort Scott, 9 mi SW, Hollister Wildlife Area, 1-14-VI-2005, canopy trap (1); 14-28-VI-2005 (3); 17-30-V-2006 (2); 30-V-7-VI-2006 (1); 7-15-VI-2006 (1); 26-VI-6-VII-2006 (1). *Douglas Co.*: 22-VI-2004, Lindgren trap, S. White SCW040326 (1); 14-VII-2004, Lindgren trap, S. White, SCW040430 (1); Baldwin, 2 mi NW, 9-16-VI-2005, canopy trap (1). *Geary Co.*: 3-VI-2004, rest stop, Lindgren trap, D. Martin, DJM040179 (1). *Johnson Co.*: Shawnee, 1.2 mi N of 43rd St, nr Kansas River, 2-9-VI-2005, canopy trap (2); 9-19-VI-2005 (1); 24-VI-5-VII-2005 (3). *Sedgwick Co.*: Derby, 0.5 mi S, NE of intersection K15 and 91st St, 21-30-VI-2005, canopy trap (1).

#### Ohio

(voucher deposited in the MSUC)

“Ohio” (1).

#### Pennsylvania

(vouchers deposited in the MSUC and additional specimens at the Pennsylvania Department of Agriculture)

*Cumberland Co.*: Roadway Dr @ Schneider Dr, 40.229030°N, 77.111580°W, 26.VI.2009, Coll. LR Donovall (25). *Dauphin Co.*: Wildwood on Industrial Rd, 40.316325°N, -76.888783°W, 6.VIII.2009, Coll. SE Spichiger, Ex. Lindgren-EtOH (2). *Lancaster Co.*: 7031 Elizabethtown Rd, 40.182583°N, -76.498783°W, 23.VII.2009, SE Spichiger, Ex. Lindgren-BEBB/EtOH (1). *York Co.*: 400 Mundis Race Rd, 40.030170°N, -76.705330°W, 10.vi.2009, Coll. S Rebert, Ex. Lindgren-Alpha/EtOH (4).

## Key to the Scolytus Geoffroy of North America East of the Mississippi River

This key treats both sexes of Scolytus species and includes all species in America east of the Mississippi and all species known to colonize hardwoods in North America. Scolytus species are typically difficult to identify, especially females. Most current keys identify males, with females determined by association with males collected from galleries or based on a priori knowledge of the nuances of Scolytus sexual dimorphism. This key allows identification of Scolytus species regardless of the user’s familiarity with the genus. Terminology is similar to that used by Blackman (1934), Bright (1976) and Wood (1982) in their respective keys. Host records were obtained from Wood (1982) and Wood and Bright (1992) and all measurements excluding Scolytus fagi were taken from Wood (1982).

**Table:** 

1	At least one abdominal sternite with a spine	2
–	Abdominal sternites without spines	5
2(1)	Sternite 2 strongly concave, basal margin strongly produced and carinate with a median obtuse point, longitudinal median line weakly carinate on apical half; sternite 3 armed by three spines (1 median, 2 lateral) on apical margin; sternite 4 armed by a median spine on apical margin; sternite 5 with a weak transverse carina at middle of segment, apical half pubescent; frons flattened, coarsely longitudinally aciculate, frons covered with long hair-like setae; setae on lateral and dorsal margins thicker, longer, incurved. Length 2.9–5.0 mm; Carya spp.	Scolytus quadrispinosus Say male
–	Sternite 2 oblique to vertical, never concave, with a single median spine	3
3(2)	Sternite 2 armed with a weakly laterally compressed spine, bulbous apically and wider than base in male, small and quadrate in female, however the shape of the spine can be highly variable. Elytral striae and interstriae punctures equal in size. Elytra bicolored, often with a dark band. Male frons flattened, weakly longitudinally aciculate covered with hair-like setae; hair-like setae on lateral and dorsal margins thicker, longer, incurved. Female frons strongly convex, weakly aciculate, frons setae sparse, short and fine. Introduced from Asia. Length 3.0–4.0 mm; Ulmus spp.	Scolytus schevyrewi Semenov
–	Sternite 2 with a conical median spine in both sexes, elytra without a banded appearance	4
4(3)	Base of median spine reaching basal margin of sternite 2; sternites 3 and 4 with a small median tubercule on apical magins; lateral margins of sternites 2–4 with lateral teeth, sternite 5 concave with a carinate apical margin; elytral strial punctures larger than those of striae. Male frons flattened, coarsely longitudinally aciculate, abundantly covered by long hair-like setae of equal length. Female frons strongly convex, aciculate, frons setae sparse, short and fine; spine on sternite 2 smaller. Introduced from Europe. Length 1.9–3.1 mm. Ulmus spp.	Scolytus multistriatus (Marsham)
–	Base of median spine never reaching basal margin of sternite 2; lateral teeth never present on sternites; elytral strial punctures larger than those of interstriae. Male frons flat to weakly convex, moderately aciculate, abundantly covered by long hair-like setae of equal length. Female frons transversely impressed above epistoma and strongly convex above, weakly aciculate; frons setae sparse, short and fine; spine on sternite 2 smaller. Native. Length 2.2–3.3 mm; Picea spp.	Scolytus piceae (Swaine)
5(1)	Elytral interstriae and abdomen covered with very long fine hair-like setae (Scolytus muticus)	6
–	Elytral interstriae and abdomen with minute ground vestiture or with short, fine hair-like setae.	7
6(5)	Sternite 5 with a pair of strongly elevated areas on basal two-thirds, each densely covered with abundant fine, long hair-like setae, apical third strongly impressed. Sternite 2 vertical, abdomen covered in abundant fine, long hair-like setae. Elytral striae and interstriae punctures equal in size; interstriae with fine, very long hair-like setae. Frons flattened and concave, surface moderately longitudinally aciculate; hair-like setae on lateral and dorsal margins thicker, longer, incurved, remaining frons largely devoid of setae. Length 2.8–4.2 mm; Celtis occidentalis	Scolytus muticus Say male
–	Sternite 5 weakly medially concave, lacking both elevated areas on basal two-thirds and dense patches of hair-like setae. Sternite 2 vertical, abdomen covered in abundant fine, long hair-like setae. Elytral striae and interstriae punctures equal in size; interstriae with fine, very long hair-like setae. Frons less strongly flattened, nearly convex and weakly concave medially, surface finely longitudinally aciculate; hair-like setae on lateral and dorsal margins thicker, longer, incurved, remaining frons largely devoid of setae. Length 2.8–4.2 mm; Celtis occidentalis	Scolytus muticus Say female
7(5)	Sternite 2 oblique (face at an angle greater than 90° to sternite 1). Introduced species (Fig. 2a)	8
–	Sternite 2 vertical (face at an angle of approximately 90° to sternite 1). Native species (Fig. 2b)	9
8(7)	Elytral apex broadly rounded, sutural region strongly emarginate, apical margin sharply serrate, elytral interstriae with short erect setae. Sternites covered in long fine hair-like setae; sternite 5 with a weakly elevated transverse subapical carina. Pronotum coarsely, densely punctured. Male frons broadly convex, weakly impressed near median line on apical third; weakly longitudinally aciculate; lightly covered by long erect hair-like setae. Female frons more convex and covered by fewer hair-like setae. Length 1.5–2.7 mm; Crataegus spp., Cydonia spp., Malus spp., Prunus spp., Pyrus spp., Ulmus spp.	Scolytus rugulosus (Müller)
–	Elytral apex narrowly rounded, margins smooth, never obviously serrate (a row of punctures on epipleura may appear weakly serrate), elytra interstriae with short erect setae on declivity and lateral margins, surface largely glabrous. Male frons flattened, slightly impressed above epistoma, weakly convex, weakly longitudinally aciculate, frons with few short setae, setae longer more abundant on lateral margins just above epistoma; sternite 5 weakly sulcate, apical fifth rounded dorsally and moderately covered in fine hair-like setae. Female frons convex, finely aciculate with fewer hair-like setae than male; sternite 5 with a weakly elevated transverse subapical carina.Length 3.1–4.1 mm; Malus spp., Prunus spp., Pyrus spp., Ulmus spp.	Scolytus mali (Bechstein)
9(7)	Frons moderately longitudinally aciculate, with long, fine, incurved setae predominately on lateral and dorsal margins, fewer, shorter and finer setae medially. Frons nearly convex, impressed above epistoma. Sternite 5 with a moderately elevated transverse subapical carina. Elytral interstriae 1.5–2 times width of striae; elytral apex often serrate. Length 2.9–5.0 mm; Carya spp.	Scolytus quadrispinosus Say female
–	Frons with setae uniformly distributed, fewer setae on lateral and dorsal margins, shorter, finer. Frons either granulate or faintly aciculate. Elytral apex smooth. Scolytus fagi.	10
10(9)	Apical fourth of sternite 5 subvertical, transversely impressed, moderately covered with abundant fine, hair-like setae each 2–3 times the length of setae on sternite 2. Frons flattened, concave above epistoma, frons surface granulate, densely covered with fine, long setae; basal and lateral margins with fewer, shorter, finer setae. Elytral strial punctures 2–3 times the size of those on interstriae, interstriae 2–2.5 times the width of striae; surface shining, glabrous except for several short setae on declivity and lateral margins. Apex smooth. Length 3.5–6.0 mm; Celtis spp. Fagus spp.	Scolytus fagi Walsh male
–	Sternite 5 with a strongly elevated transverse subapical carina. Frons convex, weakly concave between upper level of eyes; frons faintly aciculate, devoid of granules, moderately covered with fine, long setae; basal and lateral margins with fewer, shorter, finer setae. Elytral strial punctures 2–3 times the size of those on interstriae, interstriae 2–2.5 times the width of striae; surface shining, glabrous except for several short setae on declivity and lateral margins. Apex smooth. Length 3.5–6.0 mm; Celtis spp., Fagus spp.	Scolytus fagi Walsh female
